# SAFE-CGRP study: multicenter retrospective evaluation of the safety of CGRP pathway–targeting monoclonal antibodies in migraine with relevant comorbidities or conditions excluded from trials

**DOI:** 10.3389/fneur.2025.1703876

**Published:** 2025-11-26

**Authors:** Cristina Sanabria-Gago, Iris Fernández Lázaro, Patricia Heredia, Antonio Sánchez-Soblechero, Alberto Lozano Ros, Elisa Luque Buzo, Yesica González Osorio, Ángel Guerrero-Peral, Alicia Gonzalez-Martinez, David García Azorín, Germán Latorre, Carlos Calle, Daniel Toledo-Alfocea, Javier Casas Limón, Sarai Urtiaga Valle, Marta González Salaices, Guillermo Martín Ávila, Rodrigo Terrero Carpio, Julio Pascual, Vicente González-Quintanilla, Jorge Madera, Marcos Polanco, Jaime Rodríguez-Vico, Alex Jaimes, Andrea Gómez García, María-Luz Cuadrado, Ana Beatriz Gago-Veiga

**Affiliations:** 1 Headache Unit, Department of Neurology, Hospital Universitario de la Princesa, Madrid, Spain; 2 Headache Unit, Department of Neurology, Hospital General Universitario Gregorio Marañón, Madrid, Spain; 3 Headache Unit, Department of Neurology, Hospital Clínico Universitario de Valladolid, Valladolid, Spain; 4 Departments of Neurology and Immunology, Hospital Universitario de la Princesa and Instituto de Investigación Sanitaria Princesa (IIS-Princesa), Madrid, Spain; 5 Headache Unit, Department of Neurology, Hospital Universitario Río Hortega de Valladolid, Valladolid, Spain; 6 Headache Unit, Department of Neurology, Hospital Universitario de Fuenlabrada, Fuenlabrada, Spain; 7 Headache Unit, Department of Neurology, Hospital Universitario 12 de Octubre, Madrid, Spain; 8 Headache Unit, Department of Neurology, Hospital Universitario Fundación Alcorcón, Alcorcón, Spain; 9 Headache Unit, Department of Neurology, Hospital de Torrejón, Torrejón, Spain; 10 Headache Unit, Department of Neurology, Hospital Universitario de Getafe, Getafe, Spain; 11 Headache Unit, Department of Neurology, Hospital Universitario Marqués de Valdecilla, Santander, Spain; 12 Department of Medicine and Psychiatry, University of Cantabria, Santander, Spain; 13 Headache and Other Non-Degenerative Neurological Diseases Research Group, IDIVAL, Santander, Spain; 14 Headache Unit, Department of Neurology, Hospital Universitario Fundación Jiménez Díaz, Madrid, Spain; 15 Headache Unit, Department of Neurology, Hospital Universitario Clínico San Carlos, Madrid, Spain; 16 Complutense University of Madrid, Madrid, Spain

**Keywords:** safety, calcitonin gene-related peptide, monoclonal antibodies, comorbidity, migraine, risk factors, autoimmune diseases

## Abstract

**Introduction:**

Monoclonal antibodies (mAbs) targeting calcitonin gene-related peptide (CGRP) have shown efficacy in the treatment of migraine. However, certain comorbidities have been excluded from clinical trials or may pose potential risks, despite not being formal contraindications. This study aimed to assess the safety of anti-CGRP or anti-CGRP receptor mAbs in real-world patients with such conditions.

**Methods:**

A retrospective multicenter study was conducted across 11 headache units in Spain. Patients with relevant or trial-excluded comorbidities who received at least one anti-CGRP or anti-CGRP receptor mAb were included.

**Results:**

Of 2,042 evaluated patients, 353 had at least one comorbidity. The mean treatment duration was 12.7 months [standard deviation (SD) = 8 months]. A total of 53 conditions were included: 202 had autoimmune diseases, 163 presented vascular risk factors or diseases [body mass index (BMI) > 30: 15.2%, diabetes: 5.38%, stroke/transient ischemic attack (TIA): 3.08%, cardiac ischaemic disease: 1.44%], of which 23 had moderate-to-high cardiovascular risk (Framingham scale); 23 had pulmonary diseases, 71 had a history of cancer, 12 were immunosuppressed, and 16 had other conditions. In 12% of cases, disease control was suboptimal after treatment initiation, without a causal relationship to the mAb. A possible treatment-related worsening was observed in 14 cases: four with arterial hypertension worsening, seven with Raynaud’s syndrome, two with arthritis flares, and one hereditary angioedema; all improved upon treatment discontinuation. No severe adverse events were reported.

**Discussion:**

In this study, treatment with monoclonal antibodies acting on the CGRP pathway showed a favorable safety profile in patients with complex clinical conditions not represented in clinical trials, with no serious adverse events observed during extended follow-up. These findings support their use in real-world clinical settings, although further studies are needed to confirm their long-term safety.

## 1 Introduction

Migraine is a common and disabling neurological disorder, with a prevalence ranging between 11% and 15% (1, 2). It is the leading cause of disability among individuals under 50 years of age (3) and the second-highest contributor to years lived with disability worldwide (4).

Effective therapeutic management of acute attacks and preventive treatment is essential to improving patients’ quality of life. Since 2018, mAbs targeting the calcitonin gene-related peptide (CGRP) pathway, either the peptide itself (galcanezumab, fremanezumab, eptinezumab) or anti-CGRP receptor (erenumab), have been approved by the Food and Drug Administration (FDA) and the European Medicines Agency (EMA) as specific preventive treatments for migraine (5, 6). These agents represent a highly specific therapeutic option that has significantly improved the lives of many patients (7), with consistent evidence supporting their efficacy and favorable tolerability and safety profiles (8, 9). They have been shown to reduce both the frequency and intensity of migraine attacks (10, 11).

CGRP is a 37-amino acid neuropeptide with two isoforms: *α*-CGRP, predominant in the central and peripheral nervous systems, and *β*-CGRP, mainly expressed in the gastrointestinal tract and immune system (12, 13). Beyond the trigeminal system, CGRP is widely distributed across several organs, including the cardiovascular system, thyroid and adrenal glands, gastrointestinal tract, pituitary gland, pancreas, kidneys, bones, skin, and skeletal muscles (14).

Randomized clinical trials have demonstrated the efficacy, safety, and tolerability of these treatments (15–18). However, an important subset of patients, those with cardiovascular risk factors, high body mass index (BMI > 40 kg/m^2^), uncontrolled hypertension, previous cardiac or cerebrovascular events, active or past cancer, uncontrolled psychiatric illness, immunodeficiency, or autoimmune diseases, were excluded from these studies. Investigators were also allowed to exclude patients with any clinically significant comorbidity or laboratory abnormality (15–18).

*In vivo* studies suggest that CGRP exerts protective cardiovascular and immunomodulatory effects, contributing to vasodilation and vascular homeostasis and modulating inflammatory responses (19). This peptide plays a role in ischemia-induced vasodilation, especially in small-vessel disease (20), and its blockade could theoretically aggravate ischemic processes (21). A recent systematic review has emphasized the need to monitor blood pressure and consider potential cardiovascular risks in patients treated with these mAbs (22).

Beyond the cardiovascular system, CGRP is also involved in respiratory processes. In pulmonary diseases, endogenous CGRP appears to play a dual role: promoting inflammation and bronchial hyperreactivity in early allergic responses, but later exerting anti-inflammatory and tissue-protective effects through bronchodilation, vasodilation, and regulation of regulatory T cells (23). Therefore, CGRP blockade could theoretically attenuate these protective effects, potentially worsening conditions characterized by airway hyperreactivity (24). Preclinical evidence suggests that endogenous CGRP may protect against pulmonary hypertension, vascular remodeling, and right ventricular dysfunction, particularly in the context of chronic hypoxia (21).

Moreover, CGRP plays a direct role in immune system regulation, modulating the activity of macrophages, dendritic cells, mast cells, T lymphocytes, and NK cells. It regulates inflammation and antigen presentation, contributing to a balanced immune response. Therefore, systemic blockade of the CGRP pathway could potentially impair immune surveillance, leading to an inefficient response against pathogens. Patients with immunodeficiencies, autoimmune diseases, or those receiving immunosuppressive therapy rely even more on the coordinated activity of cells such as macrophages, dendritic cells, and NK cells. CGRP inhibition could further reduce their ability to combat infections, increasing their vulnerability to common or opportunistic infections. Current studies suggest that CGRP blockade does not significantly affect adaptive immunity (23); however, the available evidence is limited, and larger and longer-term studies, particularly in vulnerable populations, are needed to confirm this observation.

In addition, CGRP has been implicated in oncological processes. Nociceptor neurons in the tumor microenvironment interact with cancer-associated fibroblasts via mediators such as CGRP and nerve growth factor, modulating interleukin expression and natural killer (NK) cell activity. This suggests that CGRP signaling might influence tumor progression and immune surveillance (25).

Given the physiological role of CGRP across multiple systems and the potential implications of its blockade in adverse events that remain insufficiently explored, it is essential to investigate the use of therapies acting on the CGRP pathway, including anti-CGRP and anti-CGRP receptor mAbs, in populations traditionally excluded from pivotal clinical trials. As the clinical use of these CGRP pathway–targeting mAbs continues to expand, particularly among patients with complex or high-risk comorbidities, real-world data are urgently needed to better characterize their safety profile and guide evidence-based decision-making in everyday practice.

This study aimed to evaluate the safety profile of anti-CGRP or anti-CGRP receptor mAbs in patients with relevant comorbidities, including cardiovascular, pulmonary, oncological, or immunological conditions, providing real-world evidence on their tolerability, safety, and potential risks in vulnerable populations.

## 2 Methods

### 2.1 Study design

This was an observational, descriptive, multicenter retrospective cohort study involving 11 tertiary Headache Units across Spain, all of which are specialized referral centers for the management of migraine and complex headache disorders. The study was conducted in accordance with the STROBE recommendations.

The retrospective design may entail potential selection and reporting biases, which were mitigated by including consecutive patients and reviewing complete medical records.

### 2.2 Data collection

Upon approval by the Clinical Ethics Committee, the medical records of 2,042 patients who had received treatment with anti-CGRP or anti-CGRP receptor mAbs were reviewed.

All patients who had been treated with mAbs since their inclusion in each hospital’s pharmacy until May 2024 were included in the study.

### 2.3 Recruitment and eligibility criteria

All consecutive patients were screened for eligibility based on predefined inclusion and exclusion criteria.

Patients were included if they: (1) had migraine according to the criteria of the International Classification of Headache Disorders, 3rd Edition (ICHD-3); (2) had been treated with mAbs targeting CGRP or anti-CGRP receptor; (3) were ≥18 years (no upper age limit was established); and (4) had comorbidities with clinical profiles that had been excluded from previous clinical trials with these drugs, as well as relevant conditions or those that could pose potential risks, although not constituting formal contraindications to treatment. No minimum treatment duration was predefined; however, all patients were treated for at least the conventional three-month period required to assess or rule out clinical benefit in routine practice.

Patients were excluded if they: (1) did not meet diagnostic criteria for migraine, (2) were under 18 years of age; and (3) were lost to follow-up.

Of the 2,042 medical records reviewed of patients treated with mAbs targeting the CGRP pathway across all participating centers, 353 patients (17.3%) met the inclusion criteria and were included in the final analysis. The final dataset represents complete follow-up information for all analyzed cases.

### 2.4 Statistical analysis

Qualitative and quantitative ordinal variables were presented as frequency and percentage; quantitative continuous variables were presented as mean and standard deviation. These variables included baseline characteristics, type of mAb used, effectiveness, and safety parameters, both general and specific to each clinical condition.

The analysis was purely descriptive, as the high heterogeneity and uneven distribution of comorbidities among subgroups limited the feasibility and interpretability of inferential comparisons. The primary objective was to describe safety outcomes in real-world conditions. Effectiveness outcomes, such as ≥50% reduction in monthly headache or migraine days, were analyzed as secondary exploratory variables.

To assess the safety of these drugs, we used functionality and disease-specific clinical scales, evaluated both before and after initiating treatment with monoclonal antibodies acting on the CGRP pathway, along with changes in laboratory values. Possible treatment-related worsening was defined as any clinical or laboratory deterioration temporally associated with treatment, for which no alternative explanation was identified after clinical evaluation. Formal causality algorithms (such as WHO-UMC) were not applied, as the assessment relied on expert clinical judgment in each participating center. For pathologies without validated progression scales, data on disease progression were collected based on clinical, laboratory, and imaging criteria.

For the assessment of autoimmune diseases and immunosuppressed patients, analytical parameters were collected, including the white blood cell count with lymphocyte subpopulations, immunoglobulins, and acute phase reactants.

In the specific case of multiple sclerosis (MS), clinical worsening was assessed using the Expanded Disability Status Scale (EDSS), the most commonly used disability scale for these patients (26), and radiological progression (defined as the appearance of new T2 lesions or gadolinium-enhancing lesions on follow-up MRI compared to baseline) (27), during treatment with mAbs acting on the CGRP pathway. On the other hand, the clinical activity of inflammatory bowel disease (IBD) was measured using the Clinical Activity Index (CAI) for ulcerative colitis (28) and the Crohn’s Disease Activity Index (CDAI) before and after treatment (29).

For immunosuppressed patients, the cause of immunosuppression was recorded: immunosuppressive treatment for autoimmune disease, immunosuppressive treatment for cancer, immunosuppressive treatment related to transplantation, or immunosuppression due to HIV infection. Data on opportunistic diseases occurring after the initiation of the drug in these patients were also collected.

To assess vascular disorders, the Framingham Risk Score was used, which estimates the 10-year and lifetime risks of atherosclerotic cardiovascular disease (ASCVD), defined as coronary death or nonfatal myocardial infarction, or fatal or nonfatal stroke. The information required to estimate ASCVD risk includes age, race, sex, total cholesterol, high-density lipoprotein (HDL) cholesterol, systolic blood pressure, use of antihypertensive medication, diabetes status, and smoking status. Individuals are considered low risk with a risk of 10% or less, intermediate risk with a risk between 10% and 20%, and high risk with a risk of 20% or higher (30). In the case of strokes, event recurrence was reviewed, as well as the presence of neurological comorbidities after the initiation of mAb treatment, such as epilepsy, cognitive impairment, headache, or others. For arterial hypertension, blood pressure values before and after the start of the drug were compared, along with the involvement of target organs (eyes, kidneys, central nervous system, heart, or others). For ischemic heart disease, the severity of angina was assessed using the scale established by the Spanish Society of Cardiology (SEC) (31). Other comparative values used for obese patients included BMI before and after treatment.

Regarding pulmonary pathologies, the severity of chronic obstructive pulmonary disease (COPD) was evaluated using the Modified British Medical Research Council Dyspnea Scale (mMRC) to quantify breathlessness (32), as well as the Global Initiative for Chronic Obstructive Lung Disease (GOLD) criteria (33). In the case of asthma, forced expiratory volume in 1 s (FEV1) values were compared before and after treatment initiation.

For tumor pathology, data regarding tumor staging before and after the initiation of mAb treatment were collected.

Some subjects in the sample presented with more than one comorbidity, which were analyzed separately. Some patients were treated with more than one anti-CGRP or its receptor mAb. Patients with missing data for key variables were excluded from respective analyses.

### 2.5 Ethical considerations

The study was approved by the Ethics Committee of the Hospital Universitario de La Princesa (Approval No. 4484). The principles of the Declaration of Helsinki and current data protection regulations were followed. As this was a retrospective study, all data were anonymized and treated confidentially.

## 3 Results

Regarding the baseline characteristics of the sample (Table 1), women represented 89% of the study sample with a mean age of migraine onset of 19 years (SD = 10). The mean age at the time of the study was 51 years (SD = 12), and the mean duration of migraine was 32 years (SD = 14). Chronic migraine was present in 69% of the sample, with an average duration of 7.5 years (SD = 8). The remaining 31% had high-frequency episodic migraine.

**Table 1:** Baseline characteristics of the study sample.

Variable	Frequency/value
Female/male (%)	89/11%
Arterial hypertension (%)	24%
Dyslipidemia (%)	20%
Diabetes mellitus (%)	10%
Smoking habit (%)	16%
Risk alcohol consumption (%)	5%
Chronic migraine/high-frequency episodic migraine (%)	69/31%
Aura (%)	41%
Mean age at migraine onset (years)	19 ± 10 years
Mean current age (years)	51 ± 12 years
Mean duration of migraine (years)	32 ± 14 years
Mean time since chronification (years)	7.5 ± 8 years

Out of a total of 2,042 clinical records reviewed, a final sample of 353 patients (17.27%) was selected. Each comorbidity was considered as an independent case for analysis, resulting in a higher number of cases (487) than patients (353). Specifically, 341 patients had one comorbidity, 61 had two, 4 had three, and 3 patients presented with four conditions. A total of 53 distinct comorbidities were recorded, including vascular diseases, pulmonary disorders, tumors, and immunosuppressive conditions (Table 2).

**Table 2:** Comorbidities and their frequencies in the study sample.

Comorbility	Number of patients with the condition	Proportion of total (%) *N* = 487
Autoimmune diseases	202	41.48%
Autoimmune thyroid disease	84	17.25%
Inflammatory bowel disease (Crohn’s/Ulcerative colitis)	35	7.19%
Multiple sclerosis	16	3.29%
Raynaud’s phenomenon	12	2.46%
Rheumatoid arthritis	10	2.05%
Systemic lupus erythematosus	7	1.44%
Celiac disease	6	1.23%
Sjögren’s syndrome	5	1.03%
Psoriatic arthritis	5	1.03%
Myasthenia Gravis	3	0.62%
Ankylosing spondylitis	3	0.62%
Behçet’s disease	3	0.62%
Polymyalgia rheumatica	3	0.62%
Autoimmune hepatitis	2	0.41%
Polyarteritis nodosa	1	0.21%
Immune-mediated necrotizing myopathy	1	0.21%
Sacroiliitis	1	0.21%
Uveítis	1	0.21%
Chronic urticaria	1	0.21%
Autoimmune encephalitis	1	0.21%
Vascular disorders	54	11.09%
Ischemic/hemorrhagic stroke or TIA	15	3.08%
Systemic or cerebral venous thrombosis	9	1.85%
Arteriovenous malformations	7	1.44%
Ischemic disease in other locations	5	1.03%
Myocardial infarction	4	0.82%
Angina pectoris	3	0.62%
Wolff-Parkinson-White syndrome	3	0.62%
Preeclampsia–eclampsia	3	0.62%
Portal hypertension	2	0.41%
Brain aneurysm	2	0.41%
Aortic coarctation surgery	1	0.21%
Vascular risk factors	109	22.38%
BMI > 30	73	15.20%
Diabetes mellitus	19	5.38%
Poorly controlled hypertension	11	3.12%
von Willebrand disease	3	0.85%
Other thrombophilias	2	0.57%
Neoplastic conditions	71	14.58%
Cancer (breast, lung, prostate…)	62	12.73%
Meningioma	6	1.23%
Pituitary adenoma	3	0.62%
Pulmonary diseases	23	4.72%
Moderate-to-severe asthma	17	3.49%
Chronic obstructive pulmonary disease (COPD)	4	0.82%
Obstructive sleep apnea	2	0.41%
Immunosuppression	12	2.46%
Immunosuppressive therapy	10	2.05%
HIV-related immunosuppression	2	0.41%
Other conditions	16	3.29%
Irritable bowel syndrome (IBS)	4	0.82%
Epilepsy	4	0.82%
Glaucoma	2	0.41%
Pulmonary tuberculosis	1	0.21%
Polycystic kidney disease	1	0.21%
Williams syndrome	1	0.21%
Pineal cyst	1	0.21%
MELAS (mitochondrial encephalomyopathy, lactic acidosis, and stroke-like episodes)	1	0.21%
Congenital toxoplasmosis	1	0.21%

In total, 310/353 (87.8%) patients received only one mAb, 34/353 (9.6%) received two, 8/353 (2.3%) received three, and only one patient (0.3%) was treated with all four available mAbs.

In order of frequency, 202/487 (41.48%) cases had an autoimmune disease; 54/487 (11.09%) cases had a vascular disorder; 109/487 (22.38%) cases presented with at least one vascular risk factor (BMI > 30, diabetes mellitus, poorly controlled hypertension, or a thrombophilia); 71/487 (14.58%) cases had an oncological condition; 23/487 (4.72%) cases had a pulmonary disease; and 12/487 (2.46%) cases were immunosuppressed.

The most frequently used mAb in our sample was fremanezumab (43%), followed by galcanezumab (29%), erenumab (23%), and eptinezumab (5%) (Table 3).

**Table 3:** Type of monoclonal antibody represented in the sample.

Type of monoclonal antibody	Value
Fremanezumab	43%
Galcanezumab	29%
Erenumab	23%
Eptinezumab	5%

In terms of effectiveness, considered as a secondary and exploratory outcome (Table 4), improvement was observed in both the number of headache days (response rate of 54.6%) and the number of monthly migraine days (response rate of 60.3%).

**Table 4:** Treatment characteristics and effectiveness of Anti-CGRP or its receptor mAbs.

Treatment characteristics and effectiveness of anti-CGRP or its receptor mAbs	Value
Mean duration of monoclonal antibody treatment (months)	12.7 ± 8 months
Mean monthly headache days before treatment (last 3 months)	23 ± 7 days
Mean monthly headache days after treatment (last 3 months)	13 ± 10 days
Response rate of monthly headache days (%)	54.6%
Mean monthly migraine days before treatment (last 3 months)	16 ± 7 days
Mean monthly migraine days after treatment (last 3 months)	7.5 ± 8 days
Response rate of monthly migraine days (%)	60.30%
Pre-treatment medication overuse (%)	64%
Post-treatment medication overuse (%)	32%
Concomitant preventive medication adjustment (complete/partial/none) (%)	17/24/51%

Following treatment with mAbs acting on the CGRP pathway, only 32% of the sample met criteria for medication overuse versus 64% prior to treatment initiation. Complete withdrawal of concomitant preventive medication was achieved in 17% of patients, while a partial reduction was noted in 24% (Table 4).

Treatment discontinuation occurred in 220/487 (45.2%) cases: 149/487 (30.6%) due to lack of effectiveness, 49/487 (10.1%) due to protocol requirements (particularly during the initial years when treatment was required to be discontinued after 1year if the patient had shown improvement), and 22/487 (4.5%) due to common adverse effects, none of which were serious. Among cases who discontinued due to adverse effects, 16/487 received ligand-targeting mAbs (14 fremanezumab 225 mg, 2 galcanezumab 120 mg), and 6/487 were treated with a receptor-targeting mAb (erenumab 140 mg). None of the cases treated with eptinezumab discontinued due to adverse effects.

**Figure 1:**
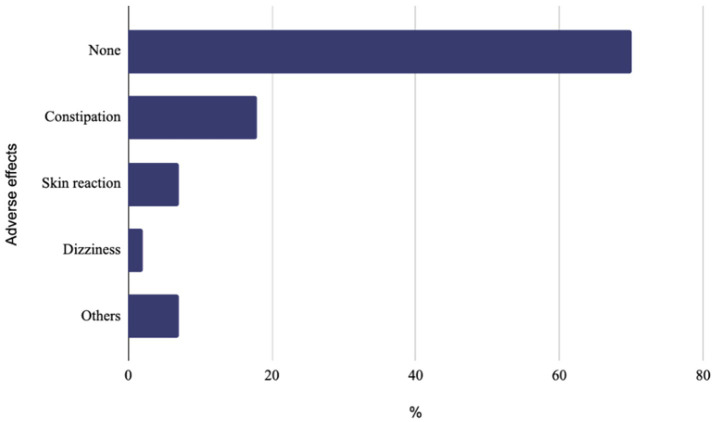
Adverse effects.

Seventy one percent (349/487) of cases did not experience any adverse effects. The most frequently reported adverse effects were constipation in 86/487 (18%) cases, local skin reactions at the injection site in 33/487 (7%), and dizziness in 19/487 (4%) cases (Figure 1).

After an average of 12.7 (SD = 8) months of treatment with anti-CGRP or its receptor mAb, 88% of comorbidities remained controlled. In the remaining 12%, poor control was not linked to the mAb. Among 173 cases with recorded data on clinical worsening following treatment initiation, only 14/173 (8%) cases showed deterioration of their comorbid condition potentially related to the therapy (Table 5).

**Table 5:** General variables related to the safety of the monoclonal antibody.

Current status of comorbidity (*n* = 487)	
Controlled	439 cases (88%)
Uncontrolled	58 cases (12%)
Clinical status of the comorbidity after treatment initiation (*n* = 173)	
No worsening	159 cases (92%)
Worsening after treatment initiation	14 cases (8%)

### 3.1 Vascular and cardiovascular findings

A total of 88/487 (18.08%) cases had arterial hypertension, of which only 11/487 (2.26%) cases presented with poorly controlled hypertension requiring at least two antihypertensive agents for management. Four of 487 (0.82%) cases required pharmacological adjustment due to poor blood pressure control following initiation of mAb treatment, with this worsening potentially related to the therapy. Two of these cases initially had grade 1 hypertension (140–159 and/or 90–99 mmHg), and the other two had grade 2 hypertension (160–179 and/or 100–109 mmHg).

Nineteen of 487 (3.9%) cases had diabetes mellitus. The mean glycated hemoglobin A1c level after treatment initiation was 6.4 mg/dL, compared to a baseline value of 6.2 mg/dL. Three patients had target organ damage prior to treatment onset: two renal and one cardiac.

Seventy-three of 487 (15%) cases with BMI > 30 were recorded, of which 40/73 (54.8%) patients had grade I obesity (BMI 30–34.9), 20/73 (27.4%) patients had grade II obesity (BMI 35–39.9), and 13/73 (17.6%) patients to grade III obesity (BMI ≥ 40). No complications were observed after starting the mAb.

Fifteen cases of 487 (3.08%) had prior history of cerebrovascular disorders, 12/487 (2.46%) were ischemic (4 atherothrombotic, 1 cardioembolic, 5 of unusual etiology, and 2 of undetermined etiology) and 3/487 (0.62%) hemorrhagic (2 hypertensive and 1 aneurysmal subarachnoid hemorrhage). No recurrences or neurological comorbidities such as stroke, epilepsy, headache, or cognitive decline were reported after treatment initiation.

Seven cases (1.44%) had a history of coronary artery disease (4 angina and 3 myocardial infarctions). All angina cases were stable and classified as grade I severity according to the Spanish Society of Cardiology (SEC) scale. No changes in left ventricular ejection fraction were observed after treatment initiation, nor were there any recurrences of acute myocardial infarction.

Cardiovascular risk for major events, estimated according to the Framingham Risk Score in 88 cases with complete clinical data, indicated that 65/88 (74%) cases had a low risk (<10%), 15/88 (17%) cases had moderate risk (10–19%), and 8/88 (9%) cases had high risk (≥20%). No vascular events occurred in any case.

Among nine cases of 487 (1.85%) of prior history of systemic or cerebral venous thrombosis, no progression or recurrence was observed.

### 3.2 Pulmonary comorbidities

A total of 4.72% of the sample (23/487 cases) had pulmonary diseases. Among them, 17/487 (3.49%) cases suffered from moderate-to-severe asthma, and 2/487 (0.41%) cases had severe obstructive sleep apnea, with no clinical scale worsening observed following mAb administration. Additionally, 4/487 (0.82%) cases had COPD, maintaining a mild stage according to the mMRC scale and GOLD criteria. Of these, three patients were classified as grade 1 and one patient as grade 2, with no changes after treatment.

### 3.3 Neoplastic conditions

Seventy-one of 487 (14.58%) cases had prior history of neoplasia, of which 6/487 (1.23%) cases were meningiomas and 3/487 (0.62%) cases were pituitary adenomas. Furthermore, 62/487 (12.73%) cases had cancer, with the specific types and their frequency listed in Table 6.

**Table 6:** Neoplastic conditions.

Type of cancer	Number of cases
Breast	33
Thyroid	5
Ovary Endometrium	3
Lung Cervix Hodgkin lymphoma Teratoma	2
Melanoma Mucoepidermoid carcinoma of the sublingual gland Non-Hodgkin Lymphoma Mucinous papillary tumor of the pancreas Transitional cell carcinoma of the bladder Multiple myeloma Prostate adenocarcinoma Pleomorphic adenoma of the parathyroid Chronic lymphocytic leukemia Basal cell carcinoma Colorectal carcinoma Enchondroma Etc.	1

Only two (0.41% of 487) cases with ductal breast carcinoma exhibited tumor stage progression during treatment. In one case, the disease advanced from stage II to stage IV, and in the other, from stage 0 to stage II. In neither case was this progression attributed to the mAb. One death was recorded due to causes unrelated to the drug. In the remaining patients, the disease remained stable or showed improvement.

### 3.4 Autoimmune diseases

The sample included 16/487 (3.29%) cases with MS. Only 1/487 (0.21%) case experienced a relapse while receiving concomitant treatment with interferon beta-1A and Galcanezumab, as assessed by the EDSS and radiological progression.

EDSS scores were generally stable, with minimal progression noted in just three cases (0.62%), corresponding to a 1.5-point increase during treatment. One case developed an episode of oligoarthritis while on galcanezumab, which resolved after discontinuation of the drug, suggesting a possible causal relationship.

A total of 35/487 (7.19%) cases had IBD. No significant changes in clinical activity were observed in any patient, as assessed by the Clinical Activity Index for ulcerative colitis and the Crohn’s Disease Activity Index.

Only one case (0.21%) developed arthritis associated with Crohn’s disease, along with worsening hereditary angioedema, both occurring after the sixth administration of fremanezumab. These conditions were managed with immunomodulatory treatment adjustments, without requiring mAb discontinuation, which was ultimately stopped after 1 year of treatment per hospital protocol.

Among the 84 of 487 cases (17.25%) with autoimmune thyroid disease, only 8 of 487 (1.64%) showed non-clinically relevant worsening of laboratory parameters during treatment. In one patient, temporary discontinuation of fremanezumab was required due to a marked elevation of thyroid-stimulating hormone (TSH), which reached 42 mIU/L, with no clear evidence of a causal relationship with the drug.

Of the 12/487 (2.46%) cases with Raynaud’s phenomenon, 7/487 (1.44%) experienced worsening after initiating treatment with anti-CGRP or its receptor mAbs, with only one case considered secondary to the drug. In a patient with inactive Raynaud’s for 2 years, symptoms reappeared tolerably after the fourth dose of fremanezumab, improving after treatment cessation. Another patient developed symptoms after the twelfth dose of fremanezumab and was diagnosed with scleroderma; good control was achieved after switching to galcanezumab and starting nimodipine. In another case, mild Raynaud’s appeared after the third dose of galcanezumab, requiring nimodipine; subsequently, arthritis developed after the fifth dose, both resolving upon treatment discontinuation. Symptoms did not recur during a second treatment cycle.

In the remaining autoimmune conditions recorded (Table 2), no clinical or laboratory worsening was observed when comparing evaluations before and after treatment, according to the scales used.

### 3.5 Immunosuppression

Twelve of 487 (2.46%) cases were either undergoing immunosuppressive treatment or immunosuppressed due to HIV infection. No alterations were observed in the white blood cell count or lymphocyte subpopulations following treatment with mAbs acting on the CGRP pathway.

## 4 Discussion

The SAFE-CGRP study retrospectively analyzes the safety of mAbs targeting CGRP or anti-CGRP receptor in migraine patients with comorbidities typically excluded from clinical trials or considered clinically relevant. This study evaluates the safety profile of anti-CGRP or its receptor mAbs across a wide range of comorbidities, highlighting the complexity of migraine patients in real-world clinical settings. The presence of multiple comorbidities is a frequent finding in routine clinical practice, and often limits the applicability of controlled study data.

Focusing on the medication’s tolerability profile, the incidence of adverse effects in our study was low, in line with previous publications (34–36), with predominantly mild manifestations such as constipation or local skin reactions. In terms of pharmacovigilance, our study did not report any adverse effects resulting from the concomitant use of another monoclonal antibody to treat a different condition, consistent with other published studies (37). These results support a favorable safety profile for these drugs. Particularly noteworthy is the clinical stability observed over an average treatment duration of 13 months, during which comorbidities remained controlled in 88% of cases. This observed safety profile is further strengthened by the clinical heterogeneity of the cohort, which included patients with over 50 different conditions and complex medical backgrounds. Overall, our findings underscore the treatment’s safety even in patients with challenging comorbidities during routine clinical follow-up.

Of the cases for which comparative clinical or laboratory data were available before and after treatment initiation, only 2.9% (14/487) showed a worsening potentially related to the drug (Supplementary Table 1). The following were possibly linked to treatment: four cases of worsened blood pressure in hypertensive patients, two arthritis episodes, one case of worsening hereditary angioedema, six cases of worsened primary Raynaud’s disease, and one case of secondary Raynaud’s. In all these cases, symptoms were resolved with symptomatic treatment or management of the comorbidity. None of the events were classified as serious, and none required discontinuation of the mAb.

CGRP has cardioprotective effects, primarily through its role in relaxing vascular smooth muscle and promoting vasodilation. At the endothelial level, CGRP can act independently of prostacyclin and, in some tissues, its activity depends on nitric oxide. Additionally, its receptor can counteract the action of endothelin, a potent vasoconstrictor, helping to maintain a balance in vascular resistance. CGRP also interacts with other vasoactive mediators, such as norepinephrine, to regulate vasoconstriction and peripheral vascular tone. Overall, the evidence suggests that CGRP functions as a compensatory vasoregulatory mechanism, and its blockade may alter cardiovascular homeostasis and the response to vasoconstrictors (19, 38). In our study, only four patients experienced elevated blood pressure, which was controlled with antihypertensive adjustments. The worsening of seven Raynaud’s syndrome cases may be explained by the same physiological mechanism. The ASCVD risk could only be calculated in 88 of 487 cases due to incomplete clinical records (age, race, sex, total cholesterol, HDL, systolic blood pressure, use of antihypertensive medication, diabetes status, and smoking status), which may introduce selection bias. These results should therefore be interpreted with caution as an approximate estimate of cardiovascular risk, consistent with previous studies supporting the cardiovascular safety of anti-CGRP or anti-CGRP receptor mAbs (39–41).

In the subgroup of patients with autoimmune diseases, two arthritis flare-ups were detected. Current literature suggests that CGRP modulates immune responses by inhibiting microglia and macrophage activity and cytokine production. Therefore, blocking CGRP might foster a pro-inflammatory state. However, the limited worsening observed in our study, along with previous literature on the use of anti-CGRP or anti-CGRP receptor mAbs in autoimmune conditions, suggests that their use is generally safe (42). Given CGRP’s role in maintaining endothelial barrier integrity and regulating vascular permeability, its inhibition might increase fluid leakage in response to inflammatory stimuli and alter the balance of vasoactive mediators, potentially explaining the angioedema case observed during treatment.

The literature highlights CGRP’s involvement, alongside other peptides, in tumor-related processes such as cell proliferation, migration, angiogenesis, and lymphangiogenesis, suggesting it may even be a potential therapeutic target (43). Our study did not identify any relevant complications in patients with past or active cancer.

No safety issues were observed in immunosuppressed patients either, despite CGRP’s role in antiviral immune responses and modulation of immune cell activity. Although the sample size of the immunosuppressed subgroup in our study was small (*n* = 12), this finding may be particularly valuable given the limited existing evidence and could help inform treatment decisions in immunocompromised populations (42, 44). Our findings enhance the understanding of anti-CGRP or its receptor mAb safety in vulnerable populations and underscore the need for prospective studies to confirm these findings and refine increasingly personalized treatment strategies for migraine management.

This study has several limitations, mainly related to its retrospective design, which may have affected the accuracy and consistency of the collected data. Such a design is inherently prone to various types of bias, including documentation bias (as some adverse events or comorbidity fluctuations may not have been systematically recorded) and information bias due to incomplete medical records.

In addition, the wide heterogeneity and unequal representation of patients across different comorbidity subgroups limit the robustness of statistical comparisons, restricting the analysis to a primarily descriptive level. The uneven distribution of disease severity, such as the predominance of low-risk cardiovascular cases, also limits the generalizability of the results.

Nevertheless, the large sample size, multicenter design, and inclusion of real-world patients with clinically relevant comorbidities enhance the external validity and reliability of the findings. These results provide valuable real-world insights into the safety profile of mAbs acting on the CGRP pathway in complex clinical populations, which should be confirmed through future prospective, controlled studies.

## 5 Conclusion

The SAFE-CGRP study provides relevant clinical evidence on the safety profile of monoclonal anti-CGRP or anti-CGRP receptor treatments in migraine patients with complex comorbidities traditionally excluded from clinical trials.

In this broad and heterogeneous cohort, with more than 50 pathologies represented, no serious adverse events clearly attributable to treatment were observed over more than 1 year of follow-up. These findings suggest a favorable safety profile in real-world conditions; however, prospective, controlled, and registry-based studies are warranted to confirm these observations and to better characterize risk profiles in specific comorbidity subgroups.

## Data Availability

The original contributions presented in the study are included in the article/Supplementary material, further inquiries can be directed to the corresponding author.
