# Supplementary material for: SAFE-CGRP study: multicenter retrospective evaluation of the safety of CGRP pathway–targeting monoclonal antibodies in migraine with relevant comorbidities or conditions excluded from trials
Source: Front Neurol. 2025 Nov 26;16:1703876. doi: 10.3389/fneur.2025.1703876 (PMC12689294; doi:10.3389/fneur.2025.1703876)
Supplement: Supplementary file 1 [file Data_Sheet_1.pdf]

Supplementary table 1. Patient Comorbidities, monoclonal antibodies, and treatment-related complications.

COMPLICATION	COMORBIDITY	mAb	DOSE (mg)	TREATMENT DURATION (months)
Worsening of blood pressure values	Poorly controlled hypertension	Galcanez umab	120	12
Worsening of blood pressure values	Poorly controlled hypertension	Galcanez umab	120	6
Worsening of blood pressure values	Poorly controlled hypertension	Fremanez umab	225	12
Worsening of blood pressure values	Poorly controlled hypertension	Fremanez umab	225	12
Arthritis flare	Crohn's disease-associated arthritis	Fremanez umab	225	10
Arthritis flare	Multiple Sclerosis	Galcanez umab	120	11
Hereditary angioedema attack	Hereditary angioedema	Fremanez umab	225	11
Exacerbation of Raynaud's syndrome	Raynaud's phenomenon	Fremanez umab	225	22
Exacerbation of Raynaud's syndrome	Raynaud's phenomenon	Galcanez umab	120	20
Exacerbation of Raynaud's syndrome	Raynaud's phenomenon	Galcanez umab	120	8
Exacerbation of Raynaud's syndrome	Raynaud's phenomenon	Galcanez umab	120	5
Exacerbation of Raynaud's syndrome	Raynaud's phenomenon	Fremanez umab	225	4
Secondary Raynaud's phenomenon	Raynaud's phenomenon	Galcanez umab	120	7
Exacerbation of Raynaud's syndrome	Raynaud's phenomenon	Fremanez umab	225	12
